# Supportive care 2030 movement: towards unifying ambitions for global excellence in supportive cancer care—an international Delphi study

**DOI:** 10.1016/j.eclinm.2024.102825

**Published:** 2024-09-11

**Authors:** Raymond Javan Chan, Reegan Knowles, Fredrick D. Ashbury, Joanne Bowen, Alexandre Chan, Melissa Chin, Ian Olver, Carolyn Taylor, Stacey Tinianov, Paola Alberti, Paolo Bossi, Norman Brito-Dellan, Tim Cooksley, Gregory Brian Crawford, Niharika Dixit, Margaret I. Fitch, Jason L. Freedman, Pamela K. Ginex, Nicolas H. Hart, Daniel L. Hertz, Michael Jefford, Bogda Koczwara, Tateaki Naito, Andrea Dahlman Orsey, Christina H. Ruhlmann, Nikolaos Tsoukalas, Corina van den Hurk, Ysabella Van Sebille, Hannah Rose Wardill, Florian Scotte, Maryam Lustberg, Multinational Association of Supportive Care in Cancer (MASCC) Study Group Chairs

**Affiliations:** a Caring Futures Institute, College of Nursing and Health Sciences, Flinders University, Flinders Drive, Bedford Park, South Australia, Australia; b Department of Oncology, University of Calgary, Alberta, Canada; c School of Biomedicine, Faculty of Health and Medical Sciences, University of Adelaide, 230 North Terrace, Adelaide, South Australia, Australia; d Department of Clinical Pharmacy Practice, School of Pharmacy and Pharmaceutical Sciences, College of Health Sciences, University of California, 802 W Peltason Dr, Irvine, CA, United States; e Multinational Association of Supportive Care in Cancer, 16 Industrial Parkway South Unit 412, Aurora, ON L4G 0R1, Canada; f School of Psychology, Faculty of Health and Medical Sciences, University of Adelaide, 230 North Terrace, Adelaide, South Australia, Australia; g Global Focus on Cancer, 26 Hemlock Rd Suite 101, South Salem, NY 10590, United States; h Advocates for Collaborative Education, Santa Cruz, CA, United States; i School of Medicine and Surgery, University of Milano-Bicocca, Monza, Italy; j Department of Biomedical Sciences, Humanitas University, Via Rita Levi Montalcini 4, Pieve Emanuele, Milan 20072, Italy; k IRCCS Humanitas Research Hospital, via Manzoni 56, Rozzano, Milan 20089, Italy; l The University of Texas MD Anderson Cancer Center, 1515 Holcombe Blvd, Houston, TX, United States; m The Christie, Wilmslow Rd, Manchester, United Kingdom; n Northern Adelaide Local Health Network, South Australia, Australia; o Division of Hematology/Oncology, University of California, San Francisco, CA, United States; p Bloomberg Faculty of Nursing, University of Toronto, 155 College Str, Suite 130, Toronto, Ontario, Canada; q Division of Oncology, Children's Hospital of Philadelphia, 3400 Civic Center Blvd, Philadelphia, PA, United States; r State University of New York, Nicolls Rd, Stony Brook, NY, United States; s Human Performance Research Centre, INSIGHT Research Institute, Faculty of Health, University of Technology Sydney (UTS), 235 Jones Str, Ultimo, Sydney, New South Wales, Australia; t Department of Clinical Pharmacy, University of Michigan College of Pharmacy, 428 Church Str, Ann Arbor, MI, United States; u Department of Health Services Research, Peter MacCallum Cancer Centre, 305 Grattan Str, Melbourne, Victoria, Australia; v Sir Peter MacCallum Department of Oncology, University of Melbourne, 305 Grattan Str, Melbourne, Victoria, Australia; w Flinders Medical Centre, Flinders Drive, Bedford Park, South Australia, Australia; x Flinders University, Flinders Health and Medical Research Institute, Flinders Drive, Bedford Park, South Australia, Australia; y Division of Thoracic Oncology, Shizuoka Cancer Center, Nagaizumi-cho, Sunto-gun, Japan; z Center for Cancer and Blood Disorders, Connecticut Children’s Medical Center, 282 Washington Street, Hartford, CT 06106, United States; aa Department of Pediatrics, University of Connecticut School of Medicine, 263 Farmington Avenue, Farmington, CT 06030, United States; ab Department of Oncology, Odense University Hospital, Denmark - Department of Clinical Research, University of Southern Denmark, Denmark; ac Oncology Department, 401 General Military Hospital of Athens, Greece; ad R&D Department, Netherlands Comprehensive Cancer Organization (IKNL), Rijnkade 5, Utrecht 3511 LC, the Netherlands; ae University of South Australia, Level 4, Catherine Helen Spence Building, City West, Adelaide, South Australia, Australia; af The Supportive Oncology Research Group, Precision Cancer Medicine Theme, The South Australian Health and Medical Research Institute, North Terrace, Adelaide, South Australia, Australia; ag Gustave Roussy Cancer Campus, 114 rue Edouard Vaillant, Villejuif CEDEX, Paris, France; ah Smilow Cancer Hospital at Yale, 35 Park Street, Fl 1st, Suite A, New Haven, CT 06511, United States; ai Fondazione IRCCS San Gerardo dei Tintori, Monza, Italy; aj Zuckerberg San Francisco General Hospital, 1001 Potrero Ave, San Francisco, CA, United States; ak Department of Pediatrics, Perelman School of Medicine, University of Pennsylvania, 3400 Civic Center Blvd, Philadelphia, PA, United States

**Keywords:** Supportive care, Supportive oncology, Palliative care, Optimal care, Toxicity

## Abstract

**Background:**

Supportive care to ensure optimal quality of life is an essential component of cancer care and symptom control across the lifespan. Ongoing advances in cancer treatment, increasing toxicity from many novel treatment regimes, and variations in access to care and cancer outcomes across the globe and resource settings present significant challenges for supportive care delivery. To date, no overarching framework has been developed to guide supportive care development worldwide. As an initial step of the Multinational Association of Supportive Care in Cancer (MASCC) Supportive Care 2030 Movement, we developed a targeted, unifying set of ambition statements to envision the future of supportive cancer care.

**Methods:**

From September 2022 until June 2023, we used a modified Delphi methodology to develop and attain consensus about ambition statements related to supportive cancer care. Leaders of MASCC Study Groups were invited to participate in an Expert Panel for the first two Delphi rounds (and a preliminary round to suggest potential ambition statements). Patient Advocates then examined and provided input regarding the ambition statements.

**Findings:**

Twenty-seven Expert Panelists and 11 Patient Advocates participated. Consensus was attained on 13 ambition statements, with two sub-statements. The ambition statements addressed global standards for guideline development and implementation, coordinated and individualized care, dedicated supportive oncology services, self-management, needs for screening and actions, patient education, behavioral support, financial impact minimization, comprehensive survivorship care, and timely palliative care, reflecting collaboration, coordination and team-based approach across all levels.

**Interpretation:**

This study is the first to develop shared ambitions for the future of supportive cancer care on a global level. These ambition statements can facilitate a coordinated, resource-stratified, and person-centered approach and inform research, education, clinical services, and policy efforts.

**Funding:**

This project received funding support from Prof Raymond Chan’s NHMRC Investigator Grant (APP1194051).

**Research in context**

**Evidence before this study**

A number of systematic reviews reported significant unmet supportive care needs in cancer survivors. These unmet needs prevail despite the development of clinical guidelines by peak organizations, suggesting significant efforts are required to advance generation of new evidence and implementation of evidence-based, best practice. To date, there have been no established consensus statements for guiding efforts to optimize supportive care on a global level.

**Added value of this study**

This study is the first to develop shared ambitions for the future of supportive cancer care on a global level. The consensus vision articulated in the ambition statements is relevant to all stakeholders of supportive care in cancer. The statements will be used to facilitate a coordinated, resource-stratified, and person-centered approach to inform and improve clinical practice, research, education and policy efforts in the supportive care.

**Implications of all the available evidence**

Together with the existing evidence for the importance of supportive cancer care to address unmet needs, the shared ambitions developed in this study will allow for prioritization of tasks, leadership and collaboration between relevant organizations and stakeholders. In addition to inspiring change, the statements will guide ongoing evaluation of progress in the area over time.

## Introduction

Supportive cancer care to ensure quality of life for both patients and caregivers has been widely acknowledged by the cancer care community as a whole and is regarded as an essential component of comprehensive cancer care.^1^ Supportive care is defined as “the prevention and management of the adverse effects of cancer and its treatment. This includes management of physical and psychological symptoms and side effects across the continuum of the cancer trajectory from diagnosis through treatment to post-treatment care”.^1^ Cancer symptoms and side effects and toxicities of treatment can be acute or chronic and occur immediately after treatment or many years later; they can impact adherence to therapy, service utilization, and patient quality of life.^2^ Specific examples of supportive care include prophylaxis and management of symptoms such as nausea; nutritional and exercise support; psychological support; and practical support for concerns such as parking and transportation.^1^

People diagnosed with cancer may experience physical, social, psychological, emotional, and spiritual changes including pain, fatigue, fear, anxiety, depression and existential distress.^3^ Needs arising from the changes experienced during cancer vary from person to person, and even within the same individual throughout various experiences as part of the illness and afterward, e.g., at diagnosis vs during disease progression.^4^ The caregivers, friends and family of cancer patients can be charged with caring for complex cancer-related symptoms; and also experience worry, financial stress, and bereavement.^3^

Although numbers vary substantially in the literature, high rates of people who have been diagnosed with cancer experience a range of unmet needs. Up to 89% report an unmet physical need, up to 89% a psychosocial need, up to 85% report an unmet psychological need, and up to 73% have needs in activities of daily living that have not been met.^5^ People living with advanced or metastatic disease—a growing population requiring advanced treatments—also experience unique challenges of uncertainty and limited services.^6^^,^^7^ Internationally, there are also variations in health-related quality-of-life outcomes and unmet needs between countries with widely differing resources and established protocols for supportive care and cancer services.^8^

The Multinational Association of Supportive Care in Cancer (MASCC), the pre-eminent organization devoted to supportive cancer care, is committed to improving the supportive care of people with cancer via quality research, education, and clinical practice. Coordinated and evolving efforts to advance supportive care across the lifespan are required to address technological and treatment advances in cancer care, changing toxicity profiles of treatments, increasing community expectations of care and variations in healthcare infrastructure in low-, middle-, and high-income countries.

To date, there has been no consensus about supportive cancer care that can be used to guide global efforts. In 2023, the MASCC launched the Supportive Care 2030 Movement, which is devoted to developing consensus about shared ambitions regarding supportive cancer care and supporting research efforts to achieve those ambitions. The launch of this movement presented an opportune time to apply a coordinated, unifying, prioritized, and targeted approach to envision a positive future of supportive care for all people affected by cancer. Informed by experts in supportive cancer care (represented by MASCC Study Group leaders) and individuals with lived experience of cancer, the aim of this study was to develop a series of priority, consensus ambition statements to inform future practice advances and relevant research in optimizing supportive cancer care across the lifespan. Such consensus statements can inform future national and local cancer plans, research funding priorities, and the efforts of the MASCC and other cancer care organizations. The proposed statements are equitable and translatable across different geographical, cultural, and social settings.

## Methods

### Study design

**Fig. 1:**
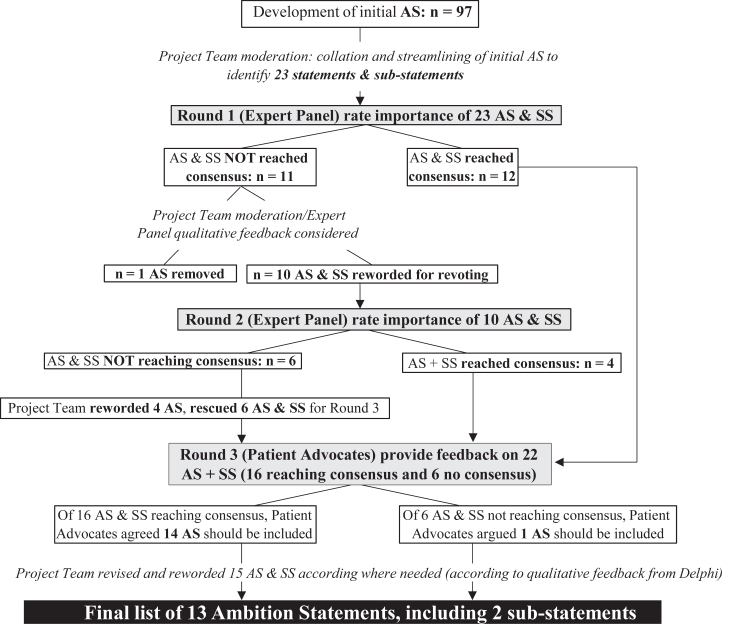
Summary of the development of the final list of ambition statements. AS, Ambition statements; SS, Sub-statements.

We used a modified Delphi methodology to achieve consensus regarding a desirable future for supportive care in cancer.^9^ Email correspondence and video conferencing was employed to accommodate participation from international experts who could provide a global perspective. We utilized the Recommendations for Conducting and Reporting of Delphi Studies (CREDES)^10^ to guide the conduct and reporting of this study. The study protocol was not prospectively registered. Fig. 1 illustrates the Delphi methodology. The Research Team included 11 investigators who are global supportive cancer care experts from the fields of medicine (n = 3), nursing (n = 1), allied health (n = 1), pharmacy (n = 1), biomedicine (n = 1), digital health (n = 1), executive leadership (n = 1), and patient advocacy (n = 2).

### Ethics

The study was reviewed and approved by the Flinders University Human Research Ethics Committee (approval code: 5636).

### Statistics

There were two Participant Groups involved in this research: the Expert Panel and the Patient Advocates. The Expert Panel consisted of MASCC Study Group (SG) leaders. At the time of this research, MASCC encompassed 20 SGs (including three sub-groups) addressing a range of supportive care domains (e.g., specific cancer toxicities, patient groups, cancer continuum stages, and cross-cutting groups; Table 1). Each SG is led by either a Chair, co-Chairs or a Chair and Vice-Chair who are experts in clinical practice and well-regarded leaders in their field. These individuals are responsible for leading research and coordinating leadership initiatives, including the development of guidelines and educational materials, and mentoring junior and mid-career supportive care scientists. All MASCC leaders (n = 37) were invited via email to participate as a member of the Expert Panel. We aimed to recruit as many of the MASCC leaders as possible. There is no clear indication of an appropriate sample size for Delphi studies, with the expertise and training in the area of research being most critical for stability of results.^11^ Patient Advocates were identified through members of the MASCC Patient Partners Committee and were approached via email to participate. Patient Advocates could be people affected by cancer who may have been involved in previous research and had indicated interest in being invited to participate in future research, and/or have identified themselves as Patient/Consumer Advocates representing people affected by cancer. Patient Advocates could also be people who work in an organization where their role is to advocate for people with cancer. It was predetermined by the Research Team, which includes two Patient Advocate leaders, that meaningful engagement of Patient Advocates (i.e., patients/carers) was planned as the last, independent round. This decision was made to ensure that the expected broadness and complexity of statements would not hamper meaningful engagement of Patient Advocates. It was also determined *a priori* that the Patient Advocate sample size would be approximately 10 participants. There is lack of consensus regarding the specific sample size required for qualitative data collection, but it is recommended that sample size should be study-specific, i.e., informed by study objectives and methodological and practical factors.^12^ We determined that 10 Patient Advocates would likely be sufficient to contribute sufficiently in-depth and rich data to inform the finalization of the ambition statements whilst being achievable given available resources. In addition, participant recruitment continued until ‘information power’ was achieved, i.e., when the Research Team determined the data collected was sufficient to address the research objectives.^13^ As described in the Procedure section below, participants voted to reach consensus on ambition statements to describe the desired future of supportive cancer care (i.e., study outcome). All Expert Panel and Patient Advocate participants signed a Participant Information and Consent Form to indicate their informed consent. Researcher made every attempt to maintain the anonymity of all participants by ensuring data was deidentified and stored appropriately, and no identifying data was shared beyond the Research Team.

**Table 1:** MASCC study group representation in expert panel.

MASCC study group (n = 20)	Number of chairs (including Chair, Vice-Chair & co-Chairs—all invited)	Number of chairs consented	Number chairs representing study group each study round		
			Preliminary round	Delphi round 1	Delphi round 2
Antiemetics	2	2	2	2	1
Bone and musculoskeletal	1	0	0	0	0
Cancer pain	2	1	1	1	0
Digital health	2	2	1	2	1
Education	2	2	2	2	0
Exercise oncology	2	2	1	1	1
Fatigue	2	1	1	0	0
Geriatrics	2	1	1	1	1
Hemostasis	2	2	2	2	1
Mucositis	2	2	2	2	2
Immuno-oncology	2	1	0	0	0
Neurological complications	2	2	2	2	2
Neutropenia, infection and immunosuppression	2	2	1	1	1
Nutrition and Cachexia	2	1	1	1	1
Oncodermatology	2	2	2	2	1
Oral care	1	0	0	0	0
Palliative care	2	1	1	1	1
Pediatrics	2	2	2	2	2
Psychosocial	2	1	1	1	1
Survivorship	2	2	1	2	2
Total (participants)	37 (1 participant is Chair of 2 groups)	28	24	25	18
Total study groups represented	20	18 (missing = Bone & musculoskeletal, and Oral care)	17 (missing = Bone & musculoskeletal, Immuno-oncology, and Oral care)	16 (missing = Bone & musculoskeletal, Fatigue, Immuno-oncology, and Oral care)	14 (missing = Bone & musculoskeletal, Education, Fatigue, Immuno-oncology, and Oral care, Cancer Pain)
Response rate	NA	76% (% of number approached that consented)	86% (of consented)	89% (of consented) ∗includes 2 that did not complete prelim round, −1 that did.	64% (of consented)

### Procedure (data collection and analysis)

#### Development of the initial ambition statements (online preliminary survey—Expert Panel)

Beginning in September, 2022, each member of the Expert Panel suggested up to five ambition statements, as well as corresponding research activities that could contribute to achieving that statement, via an online survey. It was decided *a priori* that asking the Expert Panel to provide research activities for each ambition statement would keep the ambition statements practical but were not going to form part of the ambition statement. Members of the Expert Panel were encouraged to submit ambition statements that were “aspirational,” “specific,” “of substantial impact on patient outcomes,” and “measurable” while also incorporating “multinational and interdisciplinary perspectives”. Participants were encouraged to consult their SG membership in the generation of statements. Statements could be within or beyond the scope of their study group; and address any, or all, stages of the cancer care continuum and people affected by cancer across the lifespan. The ambition statements and associated research activities could either be solely within the scope of the MASCC or could require collaboration with other stakeholders.

The Research Team reviewed and curated the proposed statements to create a more streamlined list for consideration in Delphi Round 1. This was a complex and comprehensive process facilitated through a series of Research Team discussions via videoconferencing and email. First, deduplication of repeated statements was conducted by two authors (RK and RC) independently and subsequently completed by both through a discussion. Second, statements were reviewed by the same two authors and categorized into topic areas that emerged through the proposed statements. Third, the Research Team (n = 11) condensed and reviewed the ambition statements to ensure a consistent writing style.

#### Modified Delphi: Round 1 (online survey–Expert Panel)

For Delphi Round 1 (January–February, 2023), members of the Expert Panel were emailed a link to an online survey that presented the refined list of ambition statements from the preliminary survey findings. Each person was asked to rate, for each ambition statement, the appropriateness of including that statement in the final Supportive Care 2030 Movement document, on a scale of 1–5 (1 = strongly disagree; 2 = disagree; 3 = neutral; 4 = agree; and 5 = strongly agree). Members of the Expert Panel also evaluated the clarity of the ambition statement by selecting ‘yes’ or ‘no’ or ‘unsure’ that the statement/activity was clearly worded. If a participant selected ‘no’ or ‘unsure,’ they were asked to enter comments via a free-text field explaining how the clarity of the statement could be improved. The participants could also suggest additional ambition statements.

We calculated the proportions of Expert Panel participants that indicated that they agreed or strongly agreed with each statement being included in the final list was calculated. The proportion of participants selecting ‘yes’, ‘no’ or ‘unsure’ regarding the clarity of the ambition statements was also calculated. No inferential tests were conducted as they were not intended. We determined *a priori* that at least 80% was the criterion for consensus, i.e., a statement reached if the Expert Panel agreed or strongly agreed it should be included in the final list of statements.^14^ Statements that reached consensus bypassed Delphi Round 2, and were sent straight to Round 3 for feedback from Patient Advocates. Statements that did not achieve consensus were reconsidered by the Expert Panel in Round 2. Where relevant, the Research Team refined and edited these ambition statements based on qualitative feedback received via the survey free-text fields and the research activities listed in the preliminary online survey.

#### Modified Delphi: Round 2 (online survey–Expert Panel)

Statements from Round 1 that did not achieve consensus, or statements that had undergone major changes, were presented again to the Expert Panel in Delphi Round 2 (March–April, 2023). The members of the panel were asked to re-vote on the appropriateness and clarity of the statements as per Round 1. We determined *a priori* that statements for which 80% or more of the participants agreed/strongly agreed it should be included in the final list were considered to have reached consensus.^14^ The Research Team subsequently revised the statements based on qualitative feedback in Round 2.

#### Modified Delphi: Round 3 (online workshop—Patient Advocates)

In April and May, 2023 online, individual interviews with Patient Advocates were conducted by one member of the Research Team (RK). Participants were emailed a list of the statements to be discussed in the interviews. We ensured that Patient Advocates were allowed the opportunity to reflect on all statements from Rounds 1 and 2 (including those that reached consensus and those that did not).^14^ The researcher prompted discussion around each ambition statement, one-by-one. The Patient Advocates were asked, for each statement, whether or not (and why) it should be included in the final list of ambition statements, how the statements could be reworded to improve their clarity, and capacity to implement in a real-world setting. Patient Advocates were also asked to identify any supportive care topics/issues not addressed in the statements and provide any other general comments.

The sessions were video/audio-recorded, and field notes were taken by the researcher facilitating the interviews. Recordings and field notes were used to inform the Research Team’s decisions about which statements were or were not included in the final list, and wording changes required for increased clarity. Given the objective of Round 3 was to contribute to the development and finalization of the statements, (i.e., rather than to identify participants’ perspectives around supportive care in cancer), content analysis was deemed an appropriate approach to data analysis. The feedback was considered in terms of whether/how it should be applied in the finalization of statements. The process of finalization of the ambition statements was iterative and involved a series of discussions amongst the Research Team. Discussions amongst the Research Team continued via email and conferencing to iteratively develop the final list of statements. Decisions around which statements were included were predominately straightforward, given the consistency in feedback across the Patient Advocates. Nuances in wording and detail required additional discussion to reach consensus. For the purposes of presenting the Delphi Round 3 findings in the manuscript, we summarized the main topics that arose in the interviews as well as presenting which statements reached the final list and which did not, and how Round 3 findings led to changes in wording of the statements.

### Role of funding source

Funding from Professor Raymond Chan’s NHMRC Investigator Grant (APP1194051) was used to cover his time in coordinating and implementing this research.

## Results

An overview of the development of the final list of ambition statements is outlined in Fig. 1.

### Preliminary survey: development of the initial ambition statements (online survey—Expert Panel)

**Fig. 2:**
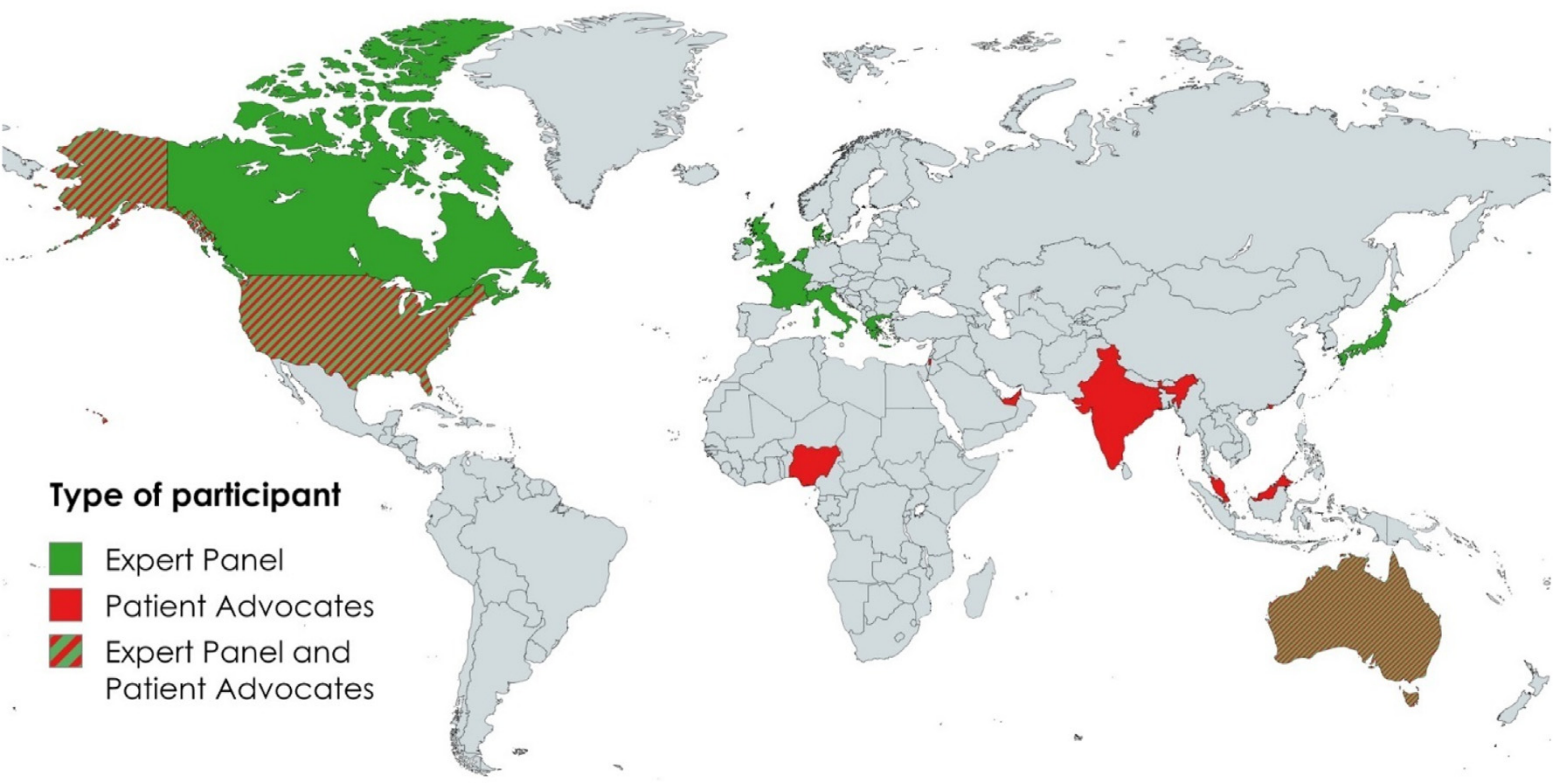
World map illustrating residence of Expert Panel and Patient Advocate study participants.

Of the 37 Chairs, co-Chairs and Vice Chairs from 20 MASCC SGs invited to participate, 28 consented to participate in the study (76%). Twenty-four leaders representing 17 different SGs participated in the preliminary survey (86% of those that consented). Only one consented individual explained why they did not participate in the preliminary survey, and this was because of a lack of internet access whilst on holiday. The Bone and Musculoskeletal, Fatigue, and Oral Care SGs were not represented in the preliminary survey. MASCC SG representation and participant demographics are shown in Tables 1 and 2, and the participants’ countries of residence are illustrated in Fig. 2. Ninety-seven ambition statements were proposed via the preliminary survey (see Supplementary File S1 for a full list of the ambition statements provided in the preliminary survey). The Research Team removed duplicated statements and this led to the removal of seven statements. To aid in further streamlining the statements, the research team members categorized the statements into 15 themes: collaboration and partnerships; comorbidities; cross-cutting/overall/holistic statements; data, technology, digital health and risk prediction; education; equitable care; exercise and nutrition; psychosocial and self-management; geriatrics; palliative care; pediatrics; patient-reported outcome measures (PROMs), patient-reported experience measures (PREMs), and needs assessment; service quality; survivorship; and toxicities and symptoms. The Research Team combined statements addressing similar topics and reworded for clarity and consistency. This process resulted in a list of 23 ambition statements for Delphi Round 1.

**Table 2:** Expert panel demographics.

Demographic	Delphi round		
	Initial statements (n = 24)	Round 1 (n = 25)	Round 2 (n = 18)
MASCC Study Groups represented (n)	17	16	14
***World region***			
The Americas	10	10	5
Asia	1	1	1
Europe	7	7	6
Oceania	7	7	6
***Years working in cancer care***			
0–9	2	2	2
10–19	11	11	9
20–29	9	9	6
30+	1	2	0
Did not report	2	1	1
***Profession/discipline***			
Medicine	15	15	11
Allied health	2	2	2
Nursing	2	3	2
Other academics	6	5	3

### Modified Delphi: Round 1 (online survey–Expert Panel)

Twenty-five participants (89% of those that consented) representing 17 different MASCC study groups completed the Round 1 online survey to rate the appropriateness and clarity of the 23 statements. The SGs not represented in Round 1 were Bone and Musculoskeletal, Fatigue, Immuno-oncology and Oral Care. One preliminary round participant did not participate in Round 1, and two people who consented but did not participate in the preliminary round, participated in Round 1. Consensus was attained (i.e., ≥80% of the participants reported they ‘agreed’ or ‘strongly agreed’ with the ambition statement being included in the final list of ambition statements) for 12 ambition statements. These statements were directed to Delphi Round 3 for the consideration of the Patient Advocates. Eleven ambition statements did not reach consensus for inclusion. Although both Round 1 and 2 surveys provided the opportunity for qualitative comments, participants chose not to provide detail regarding why they voted against including specific statements in the final list. At least 80% of respondents ‘agreed’ or ‘strongly agreed’ that seven statements were written clearly. On the basis of repeated discussions, the Research Team agreed to remove one statement that did not reach consensus. The research team made changes to statements to increase clarity (based on qualitative survey data and Research Team discussions). The 10 statements that did not reach consensus were then directed to Round 2 to be revoted upon by the Expert Panel. The results of Round 1 appear in Supplementary File S2.

### Modified Delphi: Round 2 (online survey–Expert Panel)

Eighteen participants (64% of those that consented) representing 14 MASCC Study Groups completed the Round 2 Delphi survey. The SGs not represented in Round 2 were Bone and Musculoskeletal, Education, Fatigue, Immuno-oncology, Oral Care and Pain. Participants re-rated the 10 statements that did not reach consensus in Round 1, In Round 2, four out of the 10 statements reached consensus. The research team made changes to statements to increase clarity (based on qualitative survey data and Research Team discussions). After Round 2, there were 16 ambition statements that reached consensus in either Round 1 or 2, and six that did not. The results of Round 2 appear in Supplementary File S3. All 22 ambition statements (i.e., those that did, and did not, reach consensus) advanced to Round 3, to facilitate consideration and feedback from Patient Advocates regardless of ratings by the Expert Panel.

### Modified Delphi: round 3 (individual interviews—patient advocates)

Eleven Patient Advocates (the demographics of the Patient Advocates are reported in Table 3) participated in individual online interviews; including eight people who have been diagnosed with cancer, two people who are employed in roles which involve cancer patient advocacy, and one person who provides care to two family members (one adult and one child) diagnosed with cancer. Patient Advocates provided feedback and engaged in discussion about the 16 statements that had attained consensus in Rounds 1 and 2 first, and then the six statements that did not attain consensus.

**Table 3:** Patient advocate demographics.

Demographics (n = 11)	
***Cancer type***	
Breast	6
Head & neck	1
Hodgkin’s lymphoma	1
***Type of patient advocate***	
Patient	8
Carer	1
Employed as patient advocate	2
***Years since diagnosis/became carer/advocate***	
0–9	3
10–19	2
20–29	2
30+	1
***World region***	
Africa	3
The Americas	1
Asia	4
Europe	1
Oceania	1

Patient Advocates discussed whether they believed each ambition statement should be included in the final list, and what changes were required for clarity and appropriateness. The Patient Advocates discussed the possibility of excluding two statements that had reached consensus. These statements targeted specific sub-populations within the broader cancer population (i.e., older people with cancer and young people with cancer), and Patient Advocates expressed that no one group should be emphasized in the ambition statements, as all groups are as important as one another. In contrast, Patient Advocates discussed the importance of including the ambition statement addressing financial toxicity despite it not reaching consensus in Rounds 1 and 2. Patient Advocates highlighted that financial toxicity is a significant and increasingly recognized issue for many people affected by cancer with a large magnitude of negative effect.

Synthesis of Round 3 data led to the emergence of three main priorities of Patient Advocates for the wording and content of the final list of ambition statements.

#### Priority 1: concerns about achievability and the appropriateness of the ambition statements for all settings

The Patient Advocates acknowledged the importance of setting ambitious and aspirational goals for supportive care in cancer; however, they argued that the achievability and appropriateness of some of the ambition statements would vary substantially with geographic setting according to resource availability. It is therefore critical that variations in resources and how such variations might impact ambitions, be acknowledged in the ambition statements, they noted. The participants also acknowledged that quality supportive care and research for everyone would not be achievable in low-resource settings. The final statements should clearly acknowledge and provide options for how care may need to differ according to resource availability, the Patient Advocates noted.

#### Priority 2: use of active and empowering language when referring to people affected by cancer

The Patient Advocates expressed a concern that the ambition statements under-emphasized the role of the patient in all aspects of supportive care, including education, research, and clinical care. The participants emphasized the need for the role of patient activation and the use of active and empowering language when describing the future desired state of supportive care. One Patient Advocate suggested that the term ‘receiving care’ was passive and underemphasized the role a patient should play in their own care. The Patient Advocates also argued that patients should be empowered to decide whether to engage in all aspects of care as well as research. Likewise, patients should be considered the dominant member of their own healthcare team, the Patient Advocates noted.

#### Priority 3: accessibility of ambition statements to people affected by cancer

The Patient Advocates argued that some of the statements were unclear, complicated, long, and/or repetitious. This situation accordingly reduced the statements’ accessibility. For example, multiple participants did not understand what was meant by ‘risk and resource stratification.’ The Patient Advocates furthermore identified that some of the statements were similar or repetitive. The participants suggested defining terms and shortening some of the statements. For example, describing who is included in the phrase ‘all people affected by cancer’ means statements focused on specific sub-groups (e.g., older and younger people with cancer), can be removed. Providing a definition of what ‘supportive care’ entails would also reduce the need to include descriptions in the statements, the participants highlighted.

To develop the final list of ambition statements, the Research Team considered and discussed Round 1 and 2 findings, and the recommendations and priorities of the Patient Advocates in Round 3. Two members of the Research Team (RK and RJC) prepared a draft list of ambition statements which aligned with the Patient Advocates’ preference for including 14 of the 16 statements that reached consensus, and one that did not. The Research Team at this time reworded the statements based on data from the Patient Advocates described above.

In response to Priority 1: The inclusion of the term ‘accessible’ acknowledges that supportive care options must align with a person’s setting. For example: **1. Evidence-based, guideline-driven care:** “Supportive care is *accessible* to all people affected by cancer informed by evidence-based guidelines that are promoted and *supported by the local and global community*.”

In response to Priority 2: Statements referring to people affected by cancer ‘receiving’ care were changed to ensure more active language, e.g., **9. Evidence-informed education:** “All people affected by cancer *are empowered to engage in supportive care* through the provision of evidence-informed education.” The original version of this statement indicated that people affected by cancer would *receive* evidence-informed education. In addition, the dominant role that the person affected by cancer plays in all aspects of supportive care is reflected in statement **12: Authentic collaboration:** “Meaningful and authentic collaboration among people affected by cancer, researchers, care providers, and institutions (educational, government, and non-government) informs supportive care delivery, research, and policy.”

In response to Priority 3: To improve the readability and logical flow of the overall document, two ambition statements addressing similar aspects of supportive care were combined into one main statement (screening for toxicities) and a sub-statement (collecting PROMs and PREMs). To reduce repetition and shorten the statements, a preamble and definition of common terms were also added.

**Fig. 3:**
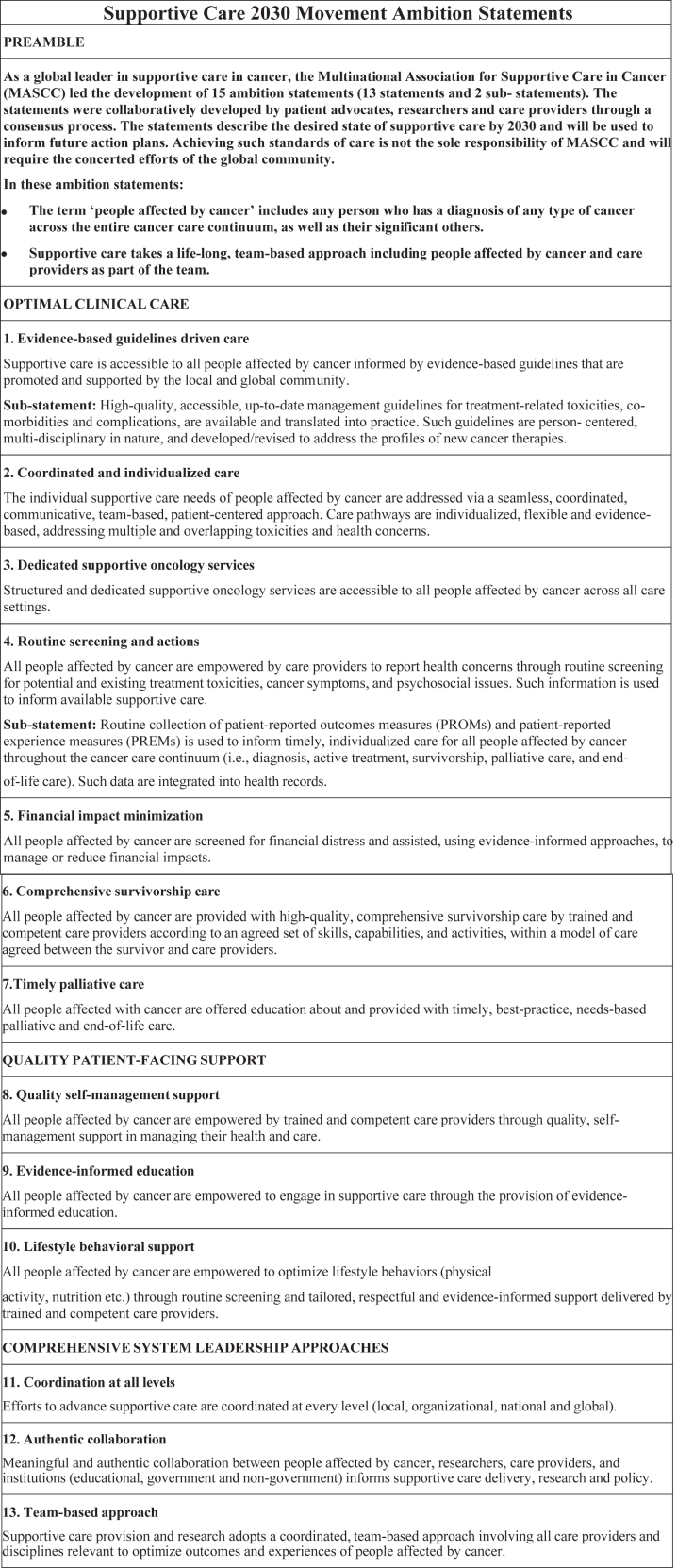
Supportive Care in Cancer 2030 Ambition Statements (with preamble) final list. MASCC, Multinational association supportive care in cancer; PROM, Patient reported outcome measures; PREM, Patient reported experience measures.

The Research Team continued to redraft the final list of ambitions through a series of in-depth and exhaustive discussions via videoconferencing and emails. The final list of statements includes 13 ambition statements (Fig. 3). Statements 1–7 primarily target optimal clinical care, statements 8–10 target quality patient-facing support, and statements 11–13 target comprehensive system leadership approaches. A preamble was developed to accompany the statements.

## Discussion

This study is the first to establish consensus on a unifying and ambitious global vision for the future of supportive care in cancer globally. The statements developed as a result of this work are relevant to all stakeholders working to optimize supportive care (e.g., people affected by cancer, care providers, researchers, and policy makers). These statements articulate a consensus vision for supportive care in cancer for the MASCC and the wider cancer care community to prioritize and lead coordinated efforts in supportive care, with the ultimate aim being to improve patient care and outcomes globally.

It is well established that patients who participate in, or self-manage, aspects of their own care exhibit superior clinical and psychosocial outcomes (e.g., improved quality of life) than patients who do not engage in self-management.^15^, ^16^, ^17^, ^18^ Patient and family caregiver engagement in research has been recognized as critical to designing studies that are relevant to the patient population^19^^,^^20^ and effectively translate research into practice.^21^ Therefore, it is important that the vision captured within these statements reflects the role of the patient in all aspects of supportive care in cancer, including acknowledging the need to better empower, activate, and support patient engagement. Several of the ambition statements explicitly reference how and when patients need to be involved in supportive care (e.g., the collection of PROMs and PREMs) and meaningful and authentic collaboration with people affected by cancer. In addition, the use of active language around patient engagement (e.g., empowerment) throughout many of the additional statements guides the development of activities where a patient’s voice is respected and encouraged. That situation empowers people affected by cancer to optimize their lifestyle behaviors and self-manage aspects of their care. Finally, we argue for the importance of continuing to involve Patient Advocates in the implementation and evaluation of these ambition statements.

The importance of equity in access of quality supportive care in cancer was a key finding from the Expert Panel and Patient Advocates. Quality supportive care is dependent on resource availability, which is variable across, and within, regions. This was reflected in our findings—participants expressed concern about the ambition statements being relevant and achievable across all settings and contexts. We advocate for being ambitious about the future of supportive care in cancer but remain realistic about what is possible given resource constraints. We advocate acknowledging and identifying the impacts of limited resources on clinical care, education, and research activities, and being innovative in how supportive care can be optimized in low-resource settings. This vision can also be supported by developing and implementing *resource-stratified* interventions and guidelines (e.g., the American Society of Clinical Oncology (ASCO)’s recently updated resource-stratified guidelines for the secondary prevention of cervical cancer^22^ and the National Comprehensive Cancer Network’s Framework for Resource Stratification.^23^ Similarly, it is critical to recognize that context and local priorities vary even in settings with similar levels of resources; such differences will in turn impact the implementation of the ambition statements.

Some participants articulated a number of means to advance towards achieving these ambitions. Such work was not part of the formal study but is valuable nonetheless. First, various nations and jurisdictions should use these specific statements to inform their cancer plans and guide the development of indicators to track their progress across various settings. Next, an audit of national cancer control plans, such as the global analysis of all aspects of planning conducted by Romero and colleagues,^24^ could be undertaken to identify current efforts to address each of the ambition statements. This could help inform the initiatives of national governments and cancer care communities across the globe. Second, the MASCC and its Study Groups should use these statements to guide their strategies and initiatives in research, education, and guideline development. It is important to acknowledge that optimizing supportive care will require the efforts of *everyone*. The MASCC will be required to partner with a variety of stakeholders (e.g., patient advocates and civil society organizations, partner organizations such as ASCO and the Union for International Cancer Control, governments, funders, and the wider clinician-researcher community) to realize its full impact. Such efforts should take into consideration the impacts of varying resource availability and other contextual factors across countries and settings. The participants also suggested that the MASCC Annual Meetings be used as an avenue to track yearly progress made towards each ambition. Such evaluation may include data-driven monitoring of service performance. It is also critical that these meetings enable inclusion of patient advocates who will continue take a key role in evaluating progress and advancing initiatives. Third, research and clinical/service improvement funders (e.g., governments and non-profit organizations) could align their funding priorities with these statements. The leaders of the MASCC can additionally actively encourage funders to consider these priorities when it comes to allocating resources.

This study is the first international investigation to develop a comprehensive and clear vision for the global future of supportive cancer care. We used the modified Delphi methodology and involved key stakeholders/experts (e.g., Patient Advocates). This methodology has been recognized as an appropriate and effective way to establish consensus.^14^ The initial item-generation phase mainly included the leaders of the MASCC SG; these individuals represent specific bespoke areas (e.g., toxicities). These SGs encompass several cross-cutting areas of importance in supportive cancer care (e.g., survivorship, education, and digital health), but we recognize that perhaps not all aspects were suitably represented. Not all SGs were represented at every round, and there was attrition of participants which may impact the findings. However, not all SGs are mutually exclusive in the work conducted and their membership, and many SG leaders have broad expertise in supportive care. Another important limitation of this research is that majority of the participants were from high-income countries; the statements that resulted from this work are clearly affected by the perspectives of the people who participated. We note that individuals from Africa were not represented in Rounds 1 and 2 of the Delphi. But three Patient Advocates in Africa were represented in Round 3. All Patient Advocates were English-speaking. Only one Patient Advocate was a carer.

The Supportive Care 2030 Movement has achieved consensus on a set of unifying, future-focused, practical ambition statements for the desired state of supportive cancer care globally by 2030. The statements encourage a consistent and collaborative approach to the development and implementation of supportive care research, education, and clinical care activities. A roadmap should be developed to provide greater detail around *when*, *what*, *how*, and by *whom* these activities should be conducted. Finally, it is critical to assess the impact of these statements. Specifically, an international audit of national cancer plans against each ambition, coordinated global research, guideline development and educational initiatives, and targeted funding opportunities are means to advance and evaluate the progress made in relation to each statement.

## Contributors

RJC, FS and ML were responsible for the conceptualization of the project. RJC, RK, FA, JB, AC, MC, IO, CT, ST, FS and ML, data curation, formal analysis, investigation, methodology and project administration. RJC and RK had full access to the data of the study. RJC supervised the project. RK wrote the original draft. All authors critically revised the manuscript for intellectual content. All authors read and approved the final version of the manuscript.

## Data sharing statement

The dataset generated from this study are available from the corresponding author on reasonable request.

## Editor note

The Lancet Group takes a neutral position with respect to territorial claims in published maps and institutional affiliations.

## Declaration of interests

All authors declare their leadership roles within the Multinational Association for Supportive Care in Cancer. The individual authors also reported their respective conflicts of interest, however are not judged to be directly relevant to the conduct of this study. FA reported reimbursement from VieCure for travel-related expenses to attend ASCO 2024 and MASCC 2024; US Patent No. 11, 798,689 B2: AI decision-support platform to generate patient-specific health plans based on unique patient circumstances; Clinical Advisory Committee Chair—VieCure; Stock—VieCure Oncology Decision Support Platform. IO reported receiving reimbursement from MASCC to travel to 2022 and 2023 MASCC Annual Scientific Meetings; and Chair of SAX Institute Board. CT reported Leadership or fiduciary roles: Executive Director, Global Focus on Cancer; Advisory Board, Asia Pacific Oncology Alliance; Steering Committee, WHO Symposium on Meaningful Engagement; Editorial Board, Cancer Survivorship Research & Care Journal. PB reported receiving consulting fees from Angelini, Nestle, Nutricia and Molteni; and participation on Angelini Advisory Board/Data Safety Monitoring Board. TN reported receiving research funding from Otsuka Pharmaceutical CO, Ltd, and Kracie Ltd; and lecture fee for ONO Pharmaceutical CO Ltd. ADO reported Pfizer stock ownership. HW reported she is the recipient of a Hospital Research Foundation Research Fellowship. FS reported payment or honoraria for lectures, presentations, speakers bureaus, manuscript writing or educational events from BMS, Sanofi, Roche, MSD, Prostrakan, Leo pharma, Janssen, Pfizer, Amgen, Pierre Fabre Oncologie, Pharmanovia, Vifor Pharma, GSK, Viatris, Helsinn, Gilead, and Daichy Sankyo. PA reported receiving grant (250.000 euros) from Cariplo Foundation; consulting fees from GHD Healthcare, Oncosultions, LLC, and Techspert. IO LTD.
