# Supplementary material for: Supportive care 2030 movement: towards unifying ambitions for global excellence in supportive cancer care—an international Delphi study
Source: eClinicalMedicine. 2024 Sep 11;76:102825. doi: 10.1016/j.eclinm.2024.102825 (PMC11415959; doi:10.1016/j.eclinm.2024.102825)
Supplement: Supplementary File S1 [file mmc1.docx]

**SUPPLEMENTARY FILE 1: AMBITION STATEMENTS PRELIMINARY ROUND**

| Acute emergency care for cancer therapy toxicities should be delivered in a personalised and sustainable manner. |
| --- |
| Supportive oncology services are available to patients 24 hours a day / 7 days a week |
| Early pathogen detection in cancer patients with infection will enable optimal antimicrobial usage improving outcomes, reducing antimicrobial side effects and unnecessary antimicrobial toxicities |
| Quality of life should be considered as important as survival for cancer patients |
| Supportive care for cancer patients should be a priority in oncology departments |
| Awareness and education of cancer patients for supportive care clinical issues should be a priority in oncology |
| Awareness and education of doctors, nurses and cancer patients for cancer associated thrombosis should be a priority |
| To assert and expand the position of MASCC as the leader in supportive oncology by continuously monitoring and updating the management of all comorbidities afflicting the patient with cancer throughout the cancer continuum, from the initial diagnosis to the management of  treatment-related toxicities and complications to survivorship to end-of-life care. |
| To assert and expand MASCC’s position as the global leader in developing best practice guidelines for managing various conditions afflicting the cancer patient throughout the cancer continuum through sound, strict, unbiased, and inclusive methodology. |
| Address financial toxicities while promoting and supporting an environment of research innovation. |
| Supportive Oncology to all |
| Address health disparities now for a more equitable 2030 |
| Separate specialty within oncology with separate boards and / or certification |
| Require a structured supportive oncology program in all cancer |
| Develop a separate field of palliative oncology |
| I would like to see a uniform structure for bench research and trial design structure as a uniform process |
| Cachexia study group which differs from nutritional study group. They are definitely different |
| Excellent guidelines concordant cancer supportive care is available to all, irrespective of where they live, their insurance status, or their ability to pay. |
| Innovation in the delivery of supportive care by implementing technology supported by clinicians. |
| Patient education needs and resources will be considered when developing any MASCC projects/resources. |
| MASCC educational resources will be developed in multiple languages and to meet needs of diverse patient populations. |
| Integrating scientific discovery with clinical research to inform the next generation of supportive care advances |
| Identification of mechanisms and supportive care approaches that transcend a single toxicity or symptom |
| Establishing the foundation for personalised supportive care through risk prediction models |
| Leveraging on our global community to promote consistency in the provision of supportive care |
| Self-management support available to all people affected by cancer |
| Patient reported outcomes collected as part of routine screening for all people with cancer at key points in disease trajectory |
| People affected by cancer involved in research planning |
| Equitable care globally |
| Equity in global research in supportive care |
| CIPN treatment guidelines |
| CIPN methodological issues in clinical trials |
| CIPN preclinical research |
| Chemobrain: a focus on |
| Health economics and CIPN |
| Cancer treatment should be personalized based on an individual's risk of treatment-related toxicity, whenever possible. |
| Develop clinical research infrastructure for decentralized interventional clinical trials for prevention or treatment of cancer treatment-related toxicity. |
| Implement patient-reported outcome monitoring in clinical practice at all Comprehensive Cancer Centers. |
| All people with cancer should be offered early comprehensive Palliative care assessment |
| Psychology assessment and support for patients, families and staff should be ready available |
| The multidisciplinary cachexia board, which is internationally certified, will be available in every oncologic care hospital for all patients with advanced cancer undergoing any intervention or care. |
| The international collaborative clinical trial group conducts RCTs of combined treatment with pharmacological and non-pharmacological interventions for cancer cachexia in multiple countries. |
| Collaboration with pharmaceutical companies to develop a new pharmacological intervention combined with our standardized exercise and nutrition. |
| Predictive measures for adverse events/nausea and vomiting will be available on a growing scale. |
| Survivor ship care will be improved across all people affected by cancer. |
| MASCC has developed the most up to date and clinically relevant Immunotoxicity management guideline. |
| Coordination of care including Digital Health opportunity |
| Development and dissemination of new careers (nurse practitioner, etc) |

| Assess patients and caregivers (consumers) needs (and unmet needs) |
| --- |
| Promote a global score to assess patients needs and expectations along the cancer course. Identify predictive factors of deterioration, complications, adverse events (cancer and treatments), efficacy/unefficacy of supportive treatments |
| Social question:   - Reduce inequity - Improve care access - Improve drug access - Evaluate psychosocial impact |
| Align and deliver MASCC Care Ambition 2030 with the WHO's 2030 Rehabilitation initiative – seeking to provide a package of interventions for cancer rehabilitation available to all countries, for system-level change that will enable access to cancer rehabilitation (regardless of low or middle or high resourcing), and implement at the population level. This will suit the MASCC Exercise Oncology and MASCC Survivorship  Groups |
| Establish exercise assessment, counselling, and where feasible, referral to exercise specialists as standard of care for all people with cancer. All people with cancer, or people affected by cancer be referred to some form of exercise services (health and wellness programs) or at-home  exercise prescriptions during and after active treatment. This will need to be supported by health behaviour change theory and frameworks. Strategies may vary by each country’s resource availability, health literacy, infrastructure, and services available. |
| Provide mandatory and routine "exercise" screening and re-assessments in order to give patients specific recommendations for their stage of cancer and type of cancer, which could reduce the toxicity related to cancer treatments, and improve quality of life, and survival. |
| Establish an international standard of certification / educational program for exercise oncology trainers to be embedded within the supportive cancer care team that is suitable for all countries (beyond those who have current standards such as ACSM, BASES, CEPA, ESSA). Many  countries (e.g., Italy or Spain) lack a specific educational program for Oncology Exercise Specialists. |
| Using repeated collection of PROs, and clinical evaluations to risk-stratify people with cancer for exercise support and mental health support and referred to appropriate services using a team-care approach due to the interaction of these needs. Understanding unique components of  various cultures and socioeconomic factors worldwide. |
| Develop specific cancer type physical activity or exercise programs on the basis of tumor clinicopathologic and molecular features for international guidance - forming a type of consensus statement or guideline. |
| Explore which physical activity or exercise modality or multimodal program is most effective to reduce the risk of recurrence and/or improving tolerance to treatments and pathological response? (e.g., different types of Breast Cancer: triple negative or hormonal receptor +) |
| There will be an internationally-recognised, clear, comprehensive sets of skills, competencies and activities for all follow-up and survivorship care for cancer survivors |
| We need a thriving international collaborative that will measure, monitor and address inequities and disparities in cancer survivorship outcomes |
| "Cancer Survivorship" is (needs to be) recognised as a cancer specialty. There will be pathways for medical, nurses and health professionals  to be recognised as survivorship specialists. We need to make sure creating a speciality DOES NOT diminish care in the primary care and generalist setting as all (specialists and generalists) have the responsibilities to care for cancer survivors. |
| Across all countries (where applicable), all cancer survivors will be able to access the type of models of care that they require (specialist-led,  PCP-led, nurse-led, supported-self-management, shared-care). This will require the systems and health professionals to be prepared in delivering such care. |
| There is a Global Self-Management Platform that can empower people affected by cancer (the ones diagnosed with their carers) to support self-management across a number of diagnoses and settings |
| "Every patient starting cancer treatment should receive a systematic assessment of support care needs, including educational and management needs, in the following areas:   - medical (symptoms and comorbidities) - psychological and social (social rights and maintenance of employment) - nutrition (diet and physical activity) - oncogenatric if &gt; 80 years of age - and fightings risk factors and addictions" |
| “Systematic/Organized screening of patients with cancer for financial toxicity.” |
| Digital Health to address supportive cancer care will be informed by best-practice guidance, rather than the development of endless platforms. |
| Disparities in digital health will be at the forefront of considerations when developing guidelines or platforms for digital health in supportive care. |
| Develop Guidelines for cancer related symptoms in older cancer patients |
| Develop communication Skills training to the clinicians regards to cancer care in geriatric oncology patients and their families |
| Develop falls prevention skills training for geriatric oncology patients, their families, and clinicians. |
| Develop guidelines for management of cognitive disorders for geriatric oncology patients. |
| Development of Guidelines for the use of comprehensive geriatric assessment (CGA) to optimize supportive care in older patients with cancer |
| Establishment of Common Data Elements that can standardize collection of supportive care data across global institutions to be used for clinical and research purposes. |
| Coherent and consistent effort to place nutrition as a priority topic MASCC across study groups. |
| Reimagine organizational approach to focus more on a holistic, contextualized perspective to optimize supportive care across cancer stages and demographic settings, rather than on specific symptoms and conditions. For example: study groups on social determinants of health, systems and models of supportive care, symptom management, complementary and integrative health. |
| Increase partnerships with other professional organizations, as well as for profit organizations like home care/hospice care organizations. |
| Involve educational institutions in scientific meetings. |
| Ensuring supportive care for children is based on pediatric-specific interventions, trials, research and guidelines and not solely based on adult extrapolation |

| Recognizing all individuals affected by the child’s cancer through family-oriented and caregiver-specific support |
| --- |
| Valuing the child’s voice through establishing pediatric-specific patient-reported outcomes |
| All children with cancer live their lives to the fullest through improvement of survivorship guidelines. |
| Ensuring global consortia and collaborations for alignment of supportive care efforts |
| To ensure implementation and quality improvement in supportive care are at the forefront of supportive care efforts; without which successful projects will not be sustained |
| To routinely collect patient-reported outcomes in the long term to ascertain the late effects of treatment (applicable to all tumor types). |
| To bring together social media and digitalization of our work in a MASCC app to use for tele-education and coaching videos for patients. |
| To establish common initiatives across MASCC study groups to enhance the multidisciplinary sharing of information and knowledge on cancer and toxicities. |
| To increase visibility of MASCC among their peers as being the expert organization for supportive care in cancer. |
| To issue clinical guidelines addressing nutritional aspects of supportive cancer care, applicable to all tumor types, from the perspective of the medical oncologist. |
| We need Structured, Easy to follow, Minimal invasive and Economically efficient guidelines with firm scientific evidence. |
| We envision that supportive care in 2030 would be accessible, universal and affordable for cancer patients across the globe for achieving adequate pain control, increasing QOL, and with an holistic approach including mind body and spirit as endpoint of care. |
| We would like to see the link in scientific discovery into our routine clinical work flows to support translational research in all MASCC member-led trials. |
| We envision that there will be more and more trials where preclinical scientists and clinical researchers will meet to build the design and to define altogether the objective, as it was a great story to tell |
| To routinely collect patient-reported outcomes from diagnosis until in follow up (applicable to all tumor types). |
| Develop ways to bring more MASCC research outcomes/ proven interventions into clinical practice: valorisation (all tumor types). |
| Improved use of the multi-disciplinary nature of MASCC to improve network supportive cancer care (all tumors) |
| Sharing data within MASCC on a multidisciplinary international data platform (all tumor types) |
| Set up an education platform to share MASCC information (all tumor types) |
