# Supplementary material for: Supportive care 2030 movement: towards unifying ambitions for global excellence in supportive cancer care—an international Delphi study
Source: eClinicalMedicine. 2024 Sep 11;76:102825. doi: 10.1016/j.eclinm.2024.102825 (PMC11415959; doi:10.1016/j.eclinm.2024.102825)
Supplement: Supplementary File S2 [file mmc2.docx]

**SUPPLEMENTARY FILE 2: RESULTS OF DELPHI ROUND 1 ONLINE SURVEY WITH EXPERT PANEL**

| **Ambition Statement** | **Reached consensus? (Proportion ‘agreed’ or ‘strongly agreed’ statement is**  **included)** | **Proportion ‘agreed’ or ‘strongly agreed’ statement is clear)** | **Action** |
| --- | --- | --- | --- |
| 1. Supportive care is accessible to all people affected by cancer based on evidence-based guidelines that are promoted and supported by the global  community. | Consensus reached (92%) | 84% | Sent to Round 3 for Patient Advocate feedback |
| 1 (sub-statement 1) A risk stratification and resource stratification approach guides care delivery. | Consensus NOT reached (76%) | 48% | Project team revised, sent to Round 2 for revoting |
| 1 (sub-statement 2) High-quality, accessible, up-to-date management guidelines for treatment-related toxicities, comorbidities and complications, are available. Such guidelines are person-centred, multi-disciplinary in nature, and developed/updated according to the development of new  anticancer therapies and their specific safety profile. | Consensus reached (84%) | 76% | Sent to Round 3 for Patient Advocate feedback |
| 2. The individual supportive care needs of people affected by cancer are addressed via a seamless, coordinated, communicative, teams-based approach, where specific supportive care needs are addressed by providers with relevant expertise. Care pathways are individualised, flexible and evidence-based, addressing multiple and overlapping toxicities and health  concerns. | Consensus reached (87.5%) | 64% | Sent to Round 3 for Patient Advocate feedback |
| 3. Structured supportive oncology services are accessible to all people  affected by cancer including within oncology departments as part of acute/emergency care. | Consensus reached (84%) | 60% | Sent to Round 3 for Patient Advocate feedback |
| 4. All people affected by cancer receive quality supportive care and self- management support from trained and competent cancer care providers. | Consensus reached (80%) | 80% | Sent to Round 3 for Patient Advocate feedback |
| 5. All people affected by cancer undergo routine screening for potential and existing treatment toxicities and cancer symptoms to inform treatment and  supportive care tailored to the individual. | Consensus reached (88%) | 88% | Sent to Round 3 for Patient Advocate feedback |
| 6. All people affected by cancer receive evidence-based education about supportive care in cancer. | Consensus reached (80%) | 80% | Sent to Round 3 for Patient Advocate feedback |
| 7. All people affected by cancer can have access to participating in supportive care research as a component of routine clinical care. | Consensus NOT reached (76%) | 80% | Project team revised, sent to Round 2 for revoting |
| 8. All people affected by cancer are screened for financial toxicity and assisted, using evidence-based approaches, to manage/reduce toxicity. | Consensus NOT reached (72%) | 60% | Project team revised, sent to Round 2 for revoting |
| 9. All people affected by cancer undergo routine screening and rescreening for counselling to optimise exercise and nutrition as core components of their treatment and wellbeing. According to need, all patients have access to exercise and nutrition specialists/services who provide individually-tailored care based on evidence-informed guidelines. All cancer care providers receive nutrition and exercise training to enable basic screening and  counselling. | Consensus NOT reached (68%) | 56% | Project team revised, sent to Round 2 for revoting |
| 10. Patient-reported outcomes measures (PROMs) and patient-reported experience measures (PREMS) are collected routinely and used to inform individualised care of all people affected by cancer throughout the cancer care continuum (i.e., active treatment, survivorship, palliative care and end-  of-life care). | Consensus NOT reached (72%) | 76% | Project team revised, sent to Round 2 for revoting |
| 11. The full potential of technology, including but not limited to digital health and artificial intelligence, in the provision of individualised care,  decision making, care coordination, and risk prediction models is realised in supportive care provision. | Consensus NOT reached (64%) | 52% | Project team revised, sent to Round 2 for revoting |
| 12. The infrastructure for standardised supportive care data collection, storage and sharing has been fully developed and implemented at a global level. | Consensus NOT reached (60%) | 52% | Removed |
| 13. All people affected by cancer will be provided with high-quality, comprehensive survivorship care by trained and competent cancer care providers according to a universally agreed set of skills, competencies and  activities, within a model of care agreed in partnership between the survivor and care providers. | Consensus reached (80%) | 64% | Sent to Round 3 for Patient Advocate feedback |
| 14. All people diagnosed with cancer are offered and provided with best- practice, timely, and individualised palliative care. | Consensus reached (80%) | 68% | Sent to Round 3 for Patient Advocate feedback |
| 15. All young people with cancer, i.e., children, adolescents and young adults, and their families and caregivers, are provided with comprehensive  and effective, supportive care based on evidence and guidelines from research in which the child’s and family’s voice is valued. | Consensus reached (80%) | 84% | Sent to Round 3 for Patient Advocate feedback |
| 16. All older people with cancer are assessed for needs using validated, evidence-based comprehensive geriatric assessment and receive comprehensive tailored supportive care according to evidence-based guidelines specific to this population. All cancer care providers are trained and adequately skilled in effective communication, assessment and support  of older people with cancer. | Consensus NOT reached (72%) | 80% | Project team revised, sent to Round 2 for revoting |
| 17. Equitable supportive care is provided to vulnerable populations including, but not limited to, culturally and linguistically diverse (CALD) populations, first nations peoples, people who identify as Lesbian, Gay, Bisexual, Trans and gender diverse, Intersex, Queer and questioning (LGBTIQ), people with low literacy, people with lower socio-economic status and those experiencing job insecurity, unstable housing or homelessness, people living in regional and rural areas, people with rarer  cancer or poorer/uncertain prognoses, prisoners, and refugees/asylum seekers. | Consensus NOT reached (72%) | 64% | Project team revised, sent to Round 2 for revoting |
| 18. Supportive care provision and research forms part of all cancer or health care plans across all jurisdictions, countries or regions. Healthcare systems support quality supportive care, including primary, secondary and tertiary  care, at all levels. | Consensus NOT reached (68%) | 56% | Project team revised, sent to Round 2 for revoting |
| 19. Supportive care approaches are aligned, synergised and coordinated at a local, organisational, nationally, and globally. | Consensus NOT reached (76%) | 64% | Project team revised, sent to Round 2 for revoting |
| 20. Collaboration between people affected by cancer, researchers, clinicians, and institutions (educational, government and non-government)  to inform supportive care research, policy and care provision is an expectation of standard. | Consensus reached (84%) | 72% | Sent to Round 3 for Patient Advocate feedback |
| 21. Supportive care provision and research adopts a coordinated teams-  based approach involving all health care providers and disciplines relevant to optimise outcomes and experiences of people affected by cancer. | Consensus reached (80%) | 72% | Sent to Round 3 for Patient Advocate feedback |
