# Supplementary material for: Supportive care 2030 movement: towards unifying ambitions for global excellence in supportive cancer care—an international Delphi study
Source: eClinicalMedicine. 2024 Sep 11;76:102825. doi: 10.1016/j.eclinm.2024.102825 (PMC11415959; doi:10.1016/j.eclinm.2024.102825)
Supplement: Supplementary File S3 [file mmc3.docx]

**SUPPLEMENTARY FILE 3: RESULTS OF DELPHI ROUND 2 ONLINE SURVEY WITH EXPERT PANEL**

| **Ambition Statement** | **Reached consensus: (Proportion ‘agreed’ or ‘strongly agreed’ statement is included)** | **Proportion of respondents ‘agreed’ or ‘strongly agreed’ statement is**  **clear** | **Action** |
| --- | --- | --- | --- |
| 1 (sub-statement 1). A risk and resource stratification approach is used to plan and deliver supportive care. | Consensus NOT reached (72%) | 56% | Project team rescued and sent statement to Round 3 |
| 7. All people affected by cancer are provided with the opportunity to participate in supportive care research. | Consensus NOT reached (72%) | 76% | Project team rescued and sent statement to Round 3 |
| 8. All people affected by cancer are screened for financial distress and assisted, using evidence-informed approaches, to manage/reduce toxicity. | Consensus NOT reached (67%) | 72% | Project team revised statement and sent to Round 3 |
| 9. All people affected by cancer undergo routine screening for tailored, evidence-informed lifestyle behaviours modification support (physical activity, nutrition, smoking, alcohol) provided by trained professionals. | Consensus reached (83%) | 78% | Sent to Round 3 for Patient Advocate feedback |
| 10. Routine collection of patient-reported outcomes measures (PROMs) and patient-reported experience measures (PREMs) inform individualised care of all people affected by cancer throughout the cancer care continuum (i.e., active treatment, survivorship, palliative care and end-of-life care).  Such data is integrated with health records. | Consensus reached (83%) | 67% | Sent to Round 3 for Patient Advocate feedback |
| 11. The full potential of available technology, including but not limited to digital health and artificial intelligence, in the provision of individualised care, decision making, care coordination, and risk prediction models is maximised in supportive care provision, delivered within ethical  boundaries. | Consensus NOT reached (56%) | 39% | Project team revised statement and sent to Round 3 |
| 15. All older people with cancer are routinely assessed for needs using validated, evidence-informed screening tests (e.g., comprehensive geriatric assessment) and receive comprehensive, evidence-informed, individualised  supportive care. | Consensus reached (83%) | 89% | Sent to Round 3 for Patient Advocate feedback |
| 16. Equitable supportive care is provided to diverse populations including, but not limited to, culturally and linguistically diverse (CALD) populations, first nations peoples, LGBTQIA+ people, people with low literacy, people with lower socio-economic status and those experiencing job insecurity, unstable housing or homelessness, people living in regional and rural areas,  people with rarer cancer or poorer/uncertain prognoses, prisoners, and refugees/asylum seekers. | Consensus NOT reached (78%) | 56% | Project team revised statement and sent to Round 3 |
| 17. Commitment to continually optimise and advance supportive care  practices and research is included in all cancer or health care plans across all jurisdictions, countries, or regions. | Consensus NOT reached (72%) | 72% | Project team revised  statement and sent to Round 3 |
| 18. Efforts to advance supportive care are coordinated at every level (local organisational, national and global). | Consensus reached (83%) | 56% | Sent to Round 3 for Patient Advocate feedback |
